# Sharing Your Husband: Adult Attachment Styles and Emotional Responses of Israeli Bedouin-Arab Women to Potential Polygynous Marriage

**DOI:** 10.3390/ijerph21101281

**Published:** 2024-09-26

**Authors:** Nuzha Allassad Alhuzail, Avi Besser, Virgil Zeigler-Hill

**Affiliations:** 1 School of Social Work, Sapir Academic College, D.N. Hof Ashkleon 7915600, Israel; 2 Department of Communication Disorders, Hadassah Academic College, Jerusalem 91010, Israel; 3 Department of Psychology, Oakland University, Rochester, MI 48309, USA; zeiglerh@oakland.edu

**Keywords:** adult attachment styles, Bedouin Arab, young women, polygynous marriage

## Abstract

Polygynous marriage is prevalent among Israel’s Bedouin-Arab community, despite being explicitly banned by state law. Cultural traditions and customs permit men to take multiple wives, and Islamic teachings do not prohibit the practice. The impact of polygyny is significant, affecting women, children, and society as a whole in various ways. We examined the connections that attachment anxiety and attachment avoidance had with hypothetical responses to the potential threat of polygynous marriages in a community sample of young unmarried Israeli Bedouin-Arab women (*n* = 306). Participants were randomly assigned to imagine either a high-threat scenario (i.e., finding that their husband decided to take a second wife; *n* = 151) or a low-threat scenario (i.e., their husband would not decide to take a second wife; *n* = 155) and report their anticipated responses to these scenarios. Results showed that attachment anxiety was positively associated with anticipated negative emotional responses in the high-threat condition but not in the low-threat condition. However, neither attachment anxiety nor attachment avoidance were associated with the perceived threat of polygyny to their marriage. The findings of this study suggest that attachment styles play a significant role in shaping women’s perceptions and reactions to hypothetical polygynous marriages. Women with anxious attachment styles are more likely to experience intensified negative emotions regarding such marriages, while those with avoidant attachment styles may display greater tolerance toward polygyny. These results highlight the need for tailored interventions and support systems that take into account individual psychological profiles as well as the broader cultural context of Israeli Bedouin-Arab society.

## 1. Introduction

Polygamous marriage refers to a union where a man has two or more wives (known as polygyny), or a woman has two or more husbands (referred to as polyandry). Of these family structures, polygyny is the most common form globally [1,2]. Despite being prohibited in most societies, polygamous relationships continue to exist across various continents and among different religious groups [3]. Estimates regarding the global prevalence of polygamy vary significantly, ranging from 4% to 54% depending on the region [4,5]. Although the overall prevalence of polygyny seems to be decreasing [4,5], certain variations of the practice, particularly in developing countries, have been on the rise. This includes de facto forms of polygyny, where an individual is legally monogamous but maintains additional relationships in a socially polygynous arrangement [3].

Research suggests that polygynous marriages can have negative effects on women’s mental health [6]. Women in these unions frequently report higher levels of depressive symptoms due to a combination of social (e.g., less social support from their husbands), psychological (e.g., insecurity that accompanies sharing one’s husband with other wives), and economic factors (e.g., economic hardships involved with supporting additional wives and children [7]). In addition, women in polygynous marriages often report increased anxiety, low self-esteem, and heightened distress [8,9].

Although there are no official statistics on the prevalence of polygyny in Israel, it is widely recognized that it exists, particularly among Bedouin-Arab communities [6]. These families are primarily located in the Negev region, which is in the southern part of Israel. Typically, polygynous marriages in Israel consist of one man married to two women, although some men may have three or four wives [10]. Such arrangements often lead to decreased marital satisfaction for the wives and can be associated with various forms of abuse—sexual, physical, and emotional—perpetrated by the husband [11].

According to the Islamic religion (i.e., the “Sharia”), Muslim men are allowed to have up to four wives, provided they can equally fulfill the financial needs of all their spouses and children. This may explain why polygyny constitutes 20–36% of marriages within the Negev Bedouin-Arab population [12]. According to one study, 21.6% of Bedouins were found to be in polygynous marriages [7]. The Israel Central Bureau of Statistics has reported a polygyny rate of 18.5% among Negev Bedouins [13]. Although the Sharia allows polygyny, Israeli law prohibits all forms of polygamy and treats it as a criminal offense, carrying a potential prison sentence of up to five years as stipulated in Section 176 of the Penal Code of 1977. Despite its illegality, the prevalence of polygyny has been on the rise during recent years [14], particularly among young, well-educated men [7]. This practice may reflect patriarchal power dynamics within a diminishing Bedouin-Arab society [15].

The Bedouin culture encourages and strengthens polygynous marriages, and includes proverbs extolling the virtues of men who have more than one wife [16]. Additionally, Bedouin culture encourages individuals to feel a stronger connection to their tribes and traditional belief systems than national laws [17]. Despite being part of a society in transition, where education is increasing, Israeli Bedouin men continue to navigate a complex cultural landscape, balancing traditional values with modern societal norms [18,19,20]. In the context of Bedouin-Arab society, polygyny does not occur in a vacuum; many factors contribute to its persistence. It reflects the status of Bedouin-Arab women, who are disadvantaged and may often feel excluded as they struggle for their place in a patriarchal tribal society that maintains the superior position granted to men [21]. Additionally, the law prohibiting polygamy in Israel is not enforced, allowing Bedouin men to bypass it by marrying according to Islamic Sharia without registering the marriage with the Interior Ministry.

Among Bedouin-Arab women in the Negev, polygynous marriages have been associated with a range of adverse psychological outcomes. Wives in such arrangements may experience depressive symptoms, low self-esteem, reduced life satisfaction, feelings of loneliness, depression, somatization, phobias, anxiety, and paranoia. These effects have been documented in several studies [2,7,9]. Moreover, the dynamics within polygynous families often result in jealousy and rivalry among co-wives, as well as an unequal division of emotional and household responsibilities [22], which can lead to conflicts between co-wives and their children [23]. The shift from a monogamous to a polygynous structure has been especially traumatic for senior wives, evoking emotional reactions comparable to those seen following a divorce [24], a phenomenon known as “First Wife Syndrome” [25]. Additionally, daughters of these marriages have also found their father’s remarriage distressing, often perceiving it as a loss of their relationship with their father [21].

The experiences of Israeli Arab mothers in polygynous families have been examined through their drawings and narratives [26]. The study revealed significant emotional distress, marked by feelings of despair, anger, loneliness, helplessness, confusion, and inner turmoil. To cope with these challenges, the mothers adopted strategies to reduce stress and shield themselves from intense emotional pain. These strategies included dissociation, where they detached from painful experiences by compartmentalizing their daily lives into functional and abusive aspects, and parentification, where they sought emotional support and comfort from their children. These coping mechanisms reflect the complex dynamics within polygynous families, characterized by jealousy among co-wives and unequal distribution of emotional resources. The study underscores the importance of greater understanding and support for women in polygynous households [26]. The patriarchal structure of Arab families is well-established in the literature; however, most of this research has centered on monogamous family systems, with far less focus on Arab women in polygynous marriages [27]. Although polygyny is associated with various negative consequences, it is also viewed positively by some [28,29]. In many cultures, polygyny is seen as a way to strengthen a family’s socioeconomic stability and security [29,30]. The additional children resulting from these marriages are valued not only for their contributions to labor but also for the emotional support they provide and the potential security they offer to their parents in old age [29,30]. A larger number of children often leads to increased economic productivity for the family.

To the best of our knowledge, no research has yet explored the attitudes and emotional reactions of unmarried young Bedouin-Arab women toward the potential threat of polygynous marriages. Additionally, no studies have examined how individual differences may affect how these women cope with the stress related to the possibility of entering a polygynous union. Considering that polygynous marriage poses a significant threat to relationships, it is crucial to understand how individual differences in personality traits related to affect regulation during stressful events and life transitions shape their reactions to this threat.

Attachment theory suggests that early relationships with caregivers are internalized as mental representations of both the self and others. These representations serve as the foundation for internal working models that influence cognition, affect, and expectations in future relationships [31,32,33]. Over the past two decades, attachment theory has emerged as a crucial framework for understanding individual differences and affect regulation during negative mood experiences. Although Bartholomew and Horowitz (1991) introduced a categorical model with four distinct adult attachment styles [34], many contemporary models conceptualize adult attachment along two orthogonal dimensions: *attachment anxiety* and *attachment avoidance* (e.g., [35,36]). Attachment anxiety involves fear of rejection and abandonment, whereas attachment avoidance reflects discomfort with intimacy and closeness (e.g., [37,38]).

The present study focuses on the two-dimensional model of attachment, as these dimensions underpin categorical views of attachment. For instance, individuals with high attachment anxiety and low attachment avoidance are often described as having a preoccupied attachment style. Attachment dimensions are thought to significantly impact how individuals respond to negative life events. Extensive research has explored the roles of attachment anxiety and avoidance in emotional self-regulation and responses to distressing situations (e.g., [37,38,39]). This is particularly relevant for situations perceived as potentially threatening to interpersonal relationships. Individuals with high attachment anxiety may exhibit hyperactivation of their attachment system in response to relational threats, whereas those with high attachment avoidance may demonstrate defensive deactivation of their attachment system [40,41,42].

Hyperactivation of the attachment system involves seeking increased proximity to the attachment figure and exhibiting greater dependency and neediness while experiencing elevated negative emotions and difficulty detaching from psychological pain [42]. Mikulincer and Shaver (2007) argued that individuals with high attachment anxiety develop hyperactivation strategies due to inconsistent caregiver responsiveness during early interactions [38]. Inconsistent caregivers may reinforce persistence in proximity-seeking behavior during perceived threats. Such individuals may remain highly vigilant for potential threats, react strongly to signs of threat, and seek closeness to others when stressed [43].

Conversely, individuals with high attachment avoidance are thought to adopt deactivating strategies due to past experiences with unavailable or disapproving attachment figures. Deactivation involves distancing from the attachment figure, increasing self-reliance, and suppressing distressing thoughts [44]. This adaptive response leads individuals with high attachment avoidance to expect better outcomes by avoiding displays of neediness or vulnerability. According to Ein-Dor et al. (2011), these individuals may develop a rapid fight-or-flight schema, minimizing the significance of threats and responding in a self-protective manner, such as escaping the situation or confronting the source of the threat [43]. Consequently, individuals with high attachment avoidance are more self-focused, less likely to seek support from others, and more inclined to suppress distress-related thoughts.

The Attachment Diathesis–Stress Process Model [45] posits that attachment insecurity serves as a diathesis, leading to maladaptive responses in the face of stressful or threatening events, depending on an individual’s attachment orientation (e.g., in romantic relationships [46,47]). Research on this model has examined internal stress, particularly how anxious attachment styles can intensify stress perceptions [45,48]. When individuals with high attachment anxiety confront significant conflicts that threaten relationship stability, they tend to experience greater distress, engage in more dysfunctional behaviors, and hold more negative views of their partners and relationships. In contrast, those with lower anxiety show opposite patterns of behavior and perception [49,50]. However, these negative effects are significantly reduced when the partners of highly anxious individuals demonstrate strong commitment to the relationship [51]. Additionally, the nature of the conflict plays a critical role. During discussions about jealousy or intimacy, highly avoidant individuals exhibit less empathic accuracy, meaning they struggle to accurately understand their partner’s thoughts and feelings. In contrast, highly anxious individuals demonstrate greater empathic accuracy, but only when they are distressed and perceive a significant threat [52]. Moreover, during tense interactions, more secure (less anxious) individuals are comforted by emotional support from their partners, whereas highly avoidant individuals prefer instrumental support, which respects their need for autonomy [38,53,54]. As a result, avoidant individuals benefit most from support that allows them to maintain their independence.

In summary, when highly anxious individuals face internal stressors, they view their partners and relationships more negatively and exhibit more dysfunctional behaviors. Highly avoidant individuals, in contrast, tend to disengage emotionally, behaviorally, and cognitively. Nevertheless, high partner commitment can buffer both anxious and avoidant individuals from their negative perceptions and reactions. Secure individuals, however, generally think, feel, and behave more constructively, particularly under acute relationship stress, which helps them maintain higher levels of personal and relational well-being [38,45]. These tendencies allow secure individuals to experience greater personal and relational satisfaction [55].

MacDonald et al. (2012) found that individuals with anxious attachment experience higher levels of ambivalence in romantic relationships, primarily due to heightened perceptions of social threats [56]. This emotional distress leads to hypervigilance for rejection and a strong need for support, resulting in conflicting feelings about intimacy and fear of abandonment. Conversely, those with avoidant attachment exhibit a weaker association with ambivalence, characterized by a dampening of perceived rewards rather than an increase in perceived threats. Avoidantly attached individuals may distort their perceptions of relationship rewards downward to justify social withdrawal, reflecting deeper fears of intimacy. The findings suggest that while both attachment styles contribute to relational ambivalence, the mechanisms differ: anxious attachment is linked to increased threat perceptions, whereas avoidant attachment is associated with decreased reward perceptions [56].

It is important to note that Katz and Katz (2022) challenge traditional views of attachment theory within the context of polyamorous relationships and argue for a reevaluation of attachment theory, which has primarily been framed within a monogamous context. They contend that this framework fails to adequately account for the dynamics of polyamorous relationships, often stigmatizing individuals with insecure attachment styles as being less capable of forming deep emotional connections. This stigma is particularly problematic in the context of *Consensual Non-Monogamy* (CNM) [57]. They highlight that many individuals who practice polyamory are predominantly securely or anxiously attached. Securely attached individuals tend to communicate effectively about intimate subjects, which is crucial in polyamorous arrangements. Conversely, anxiously attached individuals may thrive in environments where intimacy is abundant, countering the notion that only avoidantly attached individuals are drawn to multiple relationships [57]. They also discuss the importance of communication patterns in polyamorous relationships. Secure attachment traits facilitate better communication and relationship stability, whereas couples with insecure attachment styles may experience negative communication patterns that complicate their relationships [57]. They advocate for a broader understanding of attachment that includes the possibility of multiple, meaningful relationships, rather than viewing attachment as limited to dyadic pair bonding.

### Overview and Predictions

Polygynous marriages are a prevalent phenomenon in certain patriarchal societies, including Bedouin-Arab society. In the Bedouin-Arab population, the topic of polygynous marriages may be influenced by various social and cultural factors. Attachment styles can impact attitudes toward and emotional reactions to the threat of polygynous marriages through the way women perceive their relationships and their emotional needs.

**H1.**  *Women with an anxious attachment style may have a strong desire for closeness and continuous approval. Therefore, they may report more negative attitudes toward polygynous marriages, where the husband’s attention is likely to be divided. High levels of anxious attachment may also be associated with negative emotional responses to the possibility of polygynous marriage (e.g., jealousy, perceived threat).*

**H2.**  *Women with an avoidant attachment style may prefer autonomy and be less sensitive to emotional closeness, potentially being more tolerant of polygynous marriages and less distressed. They may see polygyny as a way to avoid deep emotional commitment and be less concerned about sharing with their husband.*

**Figure 1:**
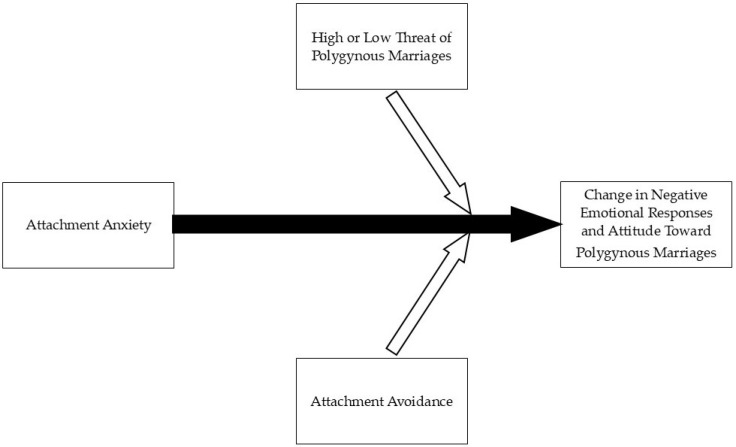
The role that attachment anxiety and attachment avoidance may play in how Israeli Bedouin-Arab women respond to hypothetical situations involving polygynous marriages.

Figure 1 presents the role of potential threat level and attachment security on changes in negative emotional responses and attitude towards polygynous marriages.

While attachment style can be a significant factor, it is important to remember that attitudes toward polygynous marriages are also regulated by social, cultural, and religious factors. Traditional gender roles, social pressure, and economic considerations may also widely influence attitudes and behaviors regarding polygyny in the Bedouin-Arab sector; however, understanding the influence of attachment styles on attitudes toward polygynous marriages can assist social initiatives and interdisciplinary research to improve intervention and enhance cultural understanding. These processes are crucial for improving the well-being of women within diverse family frameworks.

The goal of this study was to examine the attitudes and emotional responses of unmarried young Israeli Bedouin-Arab women to the potential threat of polygynous marriage, while also investigating how individual differences in attachment styles—specifically attachment anxiety and attachment avoidance—shape their perceptions and reactions.

## 2. Materials and Methods

### 2.1. Participants and Procedure

The study included a convenience sample of 324 unmarried Israeli Bedouin-Arab women, in the Negev region, who volunteered to participate by responding to requests distributed through flyers in public areas and postings on various social media platforms. We decided to restrict participation in our study to young women who were unmarried and between the ages of 16 and 24. We chose this particular age range because it represents a period in life when a significant proportion of women are expected to already be engaged due to family pressure, making the topic of marriage relevant and emotionally salient. Data from 18 participants were excluded due to their classification as univariate outliers on one or more variables. We also screened the data for issues such as cases that would be classified as multivariate outliers and indicators of inattentive responding (e.g., large inter-item standard deviations which may suggest random response patterns, long-string analysis which can identify invariant response patterns), but no cases were excluded for those reasons. We did not pre-register this study, but the data file is available on the Open Science Framework (OSF) at: https://osf.io/q9u6t/. This study had three phases: pre-manipulation, manipulation, and post-manipulation.

#### Manipulation Phase

During the pre-manipulation phase, participants were asked to complete questionnaires assessing their attachment anxiety, attachment avoidance, and current emotional state. For the manipulation phase, participants were asked to:

“*Please imagine you are married and in a committed, serious romantic relationship. You have been married for about three years, you have a small baby, and you are very happy. When you think about this romantic relationship, imagine the following scenario occur. One evening, you and your husband are sitting comfortably in the living room, the baby is asleep, and you are enjoying a quiet and peaceful evening. While sitting in the living room, you notice that your husband is reading an article in the newspaper about men in Bedouin-Arab society taking a second wife…*”

Participants were then randomly assigned to read a hypothetical scenario that was intended to invoke either high (*n* = 151) or low (*n* = 155) levels of polygynous marriage threat. The scenarios diverge at this point, with the high-threat and low-threat scenarios concluding with the following descriptions:

High-threat scenario*: Your husband says to you: “I have been thinking about men who take a second wife. It seems like a good idea. I have decided to take a second wife. It will be good for both of us and for our relationship.”*

Low-threat scenario: *Your husband says to you: “I have been thinking about men who take a second wife. It does not seem like a good idea. I would not decide to take a second wife. It would not be good for either of us or for our relationship.”*

After reading the appropriate scenario, participants were asked to provide the extent to which they would anticipate viewing polygynous marriage as a threat to their relationship with their husband and their anticipated emotional response.

### 2.2. Questionnaires

#### 2.2.1. Pre-Manipulation Measure

##### Attachment

Attachment was assessed using the Experiences in Close Relationships-Revised Questionnaire (ECR-R) [58] which includes 36 items (α = 0.81) that capture individual differences on the two major dimensions of adult attachment style: *attachment anxiety* (18 items; e.g., “I worry about being abandoned” [α = 0.88]) and *attachment avoidance* (18 items; e.g., “I am very uncomfortable being close to romantic partners” [α = 0.70]). Participants rated their level of agreement with each item using scales that ranged from 1 (*disagree strongly*) to 7 (*agree strongly*). Research has shown that this measure has adequate psychometric properties [59,60]. The Arabic version of the ECR-R has been validated within the Lebanese context. As reported in Tohme et al. (2024), researchers observed that the anxious and avoidant dimensions of the Arabic ECR-R demonstrated strong internal consistency, with alpha coefficients of 0.84 and 0.86, respectively (see [61]). Additionally, there was a reported inter-correlation of r = 0.26, *p* < 0.01 with the Arabic CES-D. Other studies have shown varying correlations between the two subscales, ranging from a minimal correlation of 0.03, and 0.05, to a higher correlation of 0.42 (see: [61]). Tohme et al. (2024) provided further support for the convergent validity of the Arabic ECR-R [61].

##### Negative Emotional State

Negative emotional states were assessed by asking participants to report their current affect (9 items; e.g., “I feel angry” [α = 0.90]). Participants rated their level of agreement with each item using scales that ranged from 1 (*disagree strongly*) to 5 (*agree strongly*).

#### 2.2.2. Post-Manipulation Measures

##### Polygynous Marriage as a Threat

Following the manipulation, participants were asked to rate the level of threat that polygyny would pose to their relationships using one item: *“To what extent would you consider your husband taking more than one wife to be a threat to your relationship with your husband?”* 1 (*not at all*) to 7 (*very much*).

##### Anticipated Negative Emotional Reaction

The anticipated negative emotional reaction of participants was assessed by asking them to report how they would anticipate feeling if they experienced the events described in the scenario (9 items; e.g., “I would be angry” [α = 0.92]). Participants rated their level of agreement with each item using scales that ranged from 1 (*disagree strongly*) to 5 (*agree strongly*).

### 2.3. Ethics Statement

Participation in this study was voluntary, and participants were aware that they could withdraw from the study at any time. All participants provided their signed, informed consent. No social security numbers or other identifying data were collected, nor were any invasive examinations conducted. This project was conducted with the approval of the Ethics Committee (IRB) of Hadassah Academic College.

### 2.4. Statistical Analysis

We began our analyses by examining the Pearson product–moment correlation coefficients among the variables. This was followed by comparing the levels of attachment anxiety, attachment avoidance, pre-manipulation negative emotional state, perception of polygynous marriage as a threat, and post-manipulation anticipated negative reaction. Finally, we conducted two hierarchical multiple regression analyses to determine whether polygynous marriage threat moderated the associations that attachment anxiety and attachment avoidance had with perceptions of polygynous marriage as a threat or anticipated negative emotional reactions. This was accomplished by regressing perception of polygynous marriage as a threat and anticipated negative emotional reactions onto attachment anxiety, attachment avoidance, polygynous threat condition (−1 = *low-threat condition*, 1 = *high-threat condition*), and pre-manipulation negative emotional state.

The continuous predictor variables were centered for the purpose of testing interactions [62]. For these analyses, the main effect terms for attachment anxiety, attachment avoidance, polygynous threat condition, and pre-manipulation negative emotional state were entered on Step 1. The following two-way interactions were included on Step 2: attachment anxiety × attachment avoidance, attachment anxiety × polygynous threat condition, and attachment avoidance × polygynous threat condition. The following three-way interaction was included on Step 3: attachment anxiety × attachment avoidance × polygynous threat condition. The regression analyses were followed by the simple slopes tests recommended to describe the interaction of continuous variables [62]. These simple slopes tests were conducted using values that were one standard deviation above or below the mean of a particular variable to represent high or low levels of that variable.

## 3. Results

The sociodemographic information for the participants can be found in Table 1. We controlled for sociodemographic variables (e.g., age, relationship status) in the preliminary analyses; however, these variables neither moderated the reported results nor significantly impacted the findings. Consequently, sociodemographic variables were excluded from the final analyses, and differences based on these variables are not discussed to maintain clarity and conciseness.

The correlation coefficients and descriptive statistics can be found in Table 2. Attachment anxiety had medium-to-very large positive correlations with pre-manipulation negative emotional state and post-manipulation anticipated negative emotional reaction in both conditions, whereas it was not correlated with attachment avoidance in either condition. Attachment anxiety had a small positive correlation with perception of polygynous marriage as a threat in the high-threat condition but not in the low-threat condition. Attachment avoidance was positively associated with pre-manipulation emotional state in the low-threat condition but not in the high-threat condition, whereas it was positively associated with post-manipulation anticipated negative emotional reaction in the high-threat condition but not in the low-threat condition. Attachment avoidance was not associated with perception of polygynous marriage as a threat in either condition.

As shown in Table 3, participants in the low-threat condition did not differ from those in the high-threat condition in terms of attachment anxiety, attachment avoidance, or pre-manipulation emotional state. These results suggest that the random assignment of participants to the low-threat and high-threat conditions led to the groups having similar levels of attachment anxiety, attachment avoidance, and pre-manipulation negative emotional states. As expected, participants in the high-threat condition reported higher levels of negative emotions than those in the low-threat condition. However, in contrast to our expectations, participants in the high-threat condition did not perceive polygynous marriage to be more of a threat to their relationship with their husband than those in the low-threat condition.

The results of the hierarchical moderated multiple regression for perception of polygynous marriage are presented in Table 4. The results revealed that there were no main effects for attachment anxiety, attachment avoidance, threat condition, or pre-manipulation negative emotional state. In addition, none of the two-way or three-way interactions were significant.

**Figure 2:**
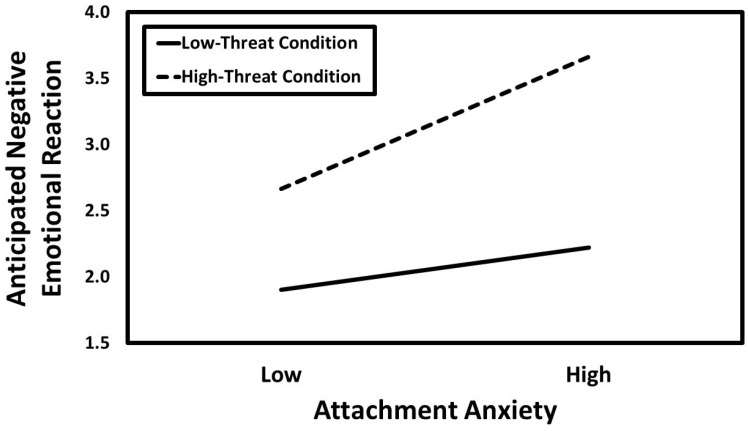
Predicted values illustrating the interactions that attachment anxiety had with the polygynous threat condition for anticipated negative emotional reaction.

The results of the hierarchical moderated multiple regression for anticipated negative emotional reaction are presented in Table 5. The results revealed positive main effects for attachment anxiety, threat condition, and pre-manipulation negative emotional state, whereas no main effect emerged for attachment avoidance. In addition, the two-way interaction of attachment anxiety × polygynous threat condition was significant. The predicted values for this interaction are presented in Figure 2. Simple slopes tests revealed that attachment anxiety was positively associated with anticipated negative emotional reaction in the high-threat condition (*B* = 0.43, *SE* = 0.08, *t* = 5.64, *p* < 0.001, *CI_95%_* [0.28, 0.58]) but not in the low-threat condition (*B* = 0.13, *SE* = 0.08, *t* = 1.75, *p* = 0.080, *CI_95%_* [−0.02, 0.28]). None of the other two-way or three-way interactions were significant.

## 4. Discussion

The aim of the present study was to examine the associations that attachment anxiety and attachment avoidance had with anticipated responses to the potential threat of polygynous marriage. Results showed that attachment anxiety was positively associated with anticipated negative emotional responses in the high-threat condition but not in the low-threat condition. As anticipated, individuals with high levels of attachment anxiety expressed the general hyperactivation effect in the high-threat condition [37]. However, neither attachment anxiety nor attachment avoidance was associated with the perceived threat of polygyny to the subject’s marriage.

In the present sample, attachment anxiety and attachment avoidance had a weak intercorrelation, which supports the assumption in most attachment theories that anxious and avoidant dimensions should be orthogonal (see e.g., [63]). For example, Mikulincer and Shaver (2003) present hyperactivating and deactivating strategies, measured by anxious and avoidant dimensions, respectively, as either/or responses to attachment-relevant threats. Thus, the assumption is that these dimensions are unrelated [37].

As in other studies in the field of attachment, it is attachment anxiety that is strongly associated with reactivity, especially in stressful situations related to romantic relationships [64]. For instance, when asked to imagine being permanently separated from their partners, highly anxious individuals exhibit particularly strong negative emotional reactions, whereas highly avoidant individuals do not [65,66]. When highly anxious individuals discuss major (but not minor) conflict topics that could destabilize their relationship, they report more distress, display more dysfunctional behaviors, and view their partners and relationships more negatively. Less anxious individuals exhibit the reverse patterns [49,50]. When highly anxious people encounter internal stressors, they perceive their partners and relationships more negatively and behave in more dysfunctional, relationship-damaging ways. Highly avoidant individuals, in contrast, disengage behaviorally, emotionally, and/or cognitively when exposed to internal stressors. Anxiously attached individuals often exaggerate their distress by constantly seeking closeness and clinging to their friends and partners to attain safety and avoid feelings of abandonment. Consequently, these individuals are continually challenged by their negative emotions, which in turn increases their unhappiness [67]. Previous findings have shown that, for example, attachment anxiety was more strongly related to depressive symptoms, whereas attachment avoidance was weakly related to depressive symptoms [68].

Conversely, the attachment avoidance dimension is organized around the deactivating emotion and behavior regulation strategy, which consists of defensive attempts to keep the attachment system downregulated to avoid being further distressed by the unavailability of an attachment figure. This strategy is characterized by extreme self-reliance, denial of attachment needs, and avoidance of emotional involvement, where the individual tries to avoid rejection from attachment figures by maintaining psychological, social, and emotional distance [38].

Although attachment theory offers valuable insights into how different attachment styles influence the emotional responses of women in polygynous marriages, integrating additional theoretical perspectives could further enhance our understanding of this phenomenon. Constructs from other theories should be included in future studies. For instance, Cognitive-Behavioral Theory (e.g., [69]) can shed light on how thought patterns contribute to anxiety and shape women’s perceptions of their roles and relationships. Social Role Theory (e.g., [70]) examines how social structures and expectations influence behavior, helping explain emotional responses grounded in gender norms within Bedouin-Arab society. Feminist Theory (e.g., [71]) is crucial for discussing the broader gender dynamics and inequalities that impact emotional well-being and attitudes toward polygynous marriage. Stress and Coping Theory (e.g., [72]) can be applied to explore how women manage stress and their emotional responses to the complexities of polygynous marriages. Additionally, Intersectionality Theory (e.g., [73]) analyzes how overlapping social identities, such as gender, ethnicity, and marital status, shape experiences and emotional responses. Incorporating these theories into future research will provide a more comprehensive understanding of the diverse experiences of women in polygynous marriages.

Some variables could potentially moderate the associations between attachment dimensions and negative emotional reactions, with cultural orientation being a significant one [68]. Cross-cultural studies indicate that the influence and impact of adult attachment on individuals’ psychosocial functioning can vary depending on their cultural background. One possible explanation for the present study’s finding that attachment anxiety had a stronger association with negative emotional responses to the threat of polygynous marriage, compared to attachment avoidance, might lie in the cultural norm hypothesis [74]. According to this hypothesis, negative emotional reactions are often incompatible with the cultural norms of the social environment. For instance, depressed individuals tend to show greater emotional reactivity in cultural contexts that emphasize emotional restraint, while they may exhibit less emotional reactivity in cultures that encourage free emotional expression [74].

Cultural differences in levels of closeness are associated with patterns of insecure attachment [67]. Attachment anxiety reflects a strong need for closeness, which is common in collectivist relational cultures, whereas attachment avoidance represents extreme self-reliance and emotional distance from others, which is more prevalent in individualistic contexts [67,75]. The Bedouin of the Negev, for instance, often find themselves torn between personal desires and tribal expectations, as well as between individualistic goals and communal commitments. The Bedouin culture is typically recognized as a traditional and collectivist society, characterized by a tribal, patriarchal social structure [18]. In accordance with the cultural norm hypothesis, this collectivist context encourages emotional control, whereas individualistic cultures promote free emotional expression [76]. Attachment anxiety is linked to emotional reactivity, whereas attachment avoidance is related to emotional suppression [77].

Based on this hypothesis, as found in the present study, in individualistic cultures, the effect size between attachment avoidance and negative emotional reactions is expected to be larger than in collectivistic cultures. Conversely, as observed in the present study, the effect size between attachment anxiety and negative emotional responses is anticipated to be smaller in individualistic cultures compared to collectivistic ones.

Although polygyny is prevalent and constitutes 20–36% of marriages within the Negev Bedouin-Arab population [7,12], and 29.6% of the current sample reported their families as being polygynous, our study demonstrated extensive resistance and non-tolerance to polygamous marriages as reported at both threat levels: 80.4% of the present sample indicated that they would consider their husbands taking more than one wife to be a significant threat to their relationship (80.8% in the high-threat and 80.0% in the low-threat conditions). One possible explanation is that Bedouin society is undergoing transformative processes, rapidly evolving from a collective and patriarchal society [78,79], with its transformation marked by increased access to higher education and integration into the labor market, resulting in a noticeable surge in entrepreneurial ventures led by Bedouin women [80]. Another possible explanation for the differential effect of threat on anticipated negative emotional responses, compared to the lack of an effect regarding perceived threat of polygyny to their marriage, might be that an attitude represents one’s stable evaluation—a predisposition—toward an object. Research in social psychology often suggests that attitudes are relatively enduring judgments regarding an object, person, or event. They can be stable over time unless strongly countered by new experiences or information [81]. Emotions, on the other hand, are characterized by their intensity and duration. They arise in response to specific stimuli and are often short-lived unless continuously triggered [82]. Thus, a major difference between an attitude and emotion is that an attitude tends to be more stable over time (stability), whereas an emotion lasts for a brief period (transient nature). It may be that the stability of the subjects’ attitudes has obscured their ability to anticipate how their views might shift if their husband suddenly announced he was taking a second wife.

It is also possible that the lack of a response to polygynous marriage as a potential threat to their relationship with their husband may have been methodological. That is, participants may not have responded to that item in the context we intended. It is possible that we were not explicit enough in directing them to consider their likely attitudes in that hypothetical situation (whereas we may have been clearer in our directions for the emotional response items). Future studies are encouraged to consider the potential need to separate general, likely stable, attitudes from state evaluations. It remains possible that stable attitudes are so strong that they overshadow the state ones.

Although this study primarily examines young women’s experiences, it does not explore the perspectives and emotional responses of men regarding polygynous marriages. Understanding the viewpoints of both genders would provide a more comprehensive view of polygamous relationships and their emotional dynamics. One potential direction for further studies might be a comparison of young men and women regarding their attitudes and emotional reactions. It would be interesting to examine the attitudes of young unmarried men and women toward polygamous marriages and explore men’s anticipated emotional responses to hypothetical scenarios in which a wife agrees or refuses to share her marriage with additional women, as well as how this relates to men’s individual differences in attachment.

While this study provides valuable insights into the relationship between attachment styles and emotional responses to polygynous marriages among young women in Bedouin-Arab culture, several limitations should be acknowledged. The sample size may limit the generalizability of the findings. Although the study included participants from a specific cultural background, a larger and more diverse sample would enhance the robustness of the results and allow for comparisons across different demographics within the Bedouin-Arab population as well as among Bedouin Arabs from different regencies.

The reliance on self-reported measures for both attachment dimensions and emotional responses may introduce biases, such as social desirability bias, where participants may respond in ways they perceive as more socially acceptable rather than their true feelings. This may also be the case for the absence of differences in levels of attitudes regarding their husbands suddenly announcing they were going to take a second wife, as they may have been responding how they thought they should respond in accordance with recent cultural norms (e.g., “a new generation of women”).

Although the present study was designed and intended to be contextualized within the Bedouin-Arab culture, which has unique societal norms and beliefs regarding marriage and family structure, and while this focus on a specific cultural context is valuable, it may limit the applicability of the results to other cultural groups where attachment styles and perceptions of polygyny might differ significantly.

Moreover, participants were asked to respond to hypothetical scenarios regarding polygynous marriages. The nature of hypothetical situations may not accurately capture the complexity and emotional depth of real-life experiences. Participants’ reactions in actual situations could differ significantly, which limits the ecological validity of the findings. In addition, the study may not have accounted for all potential confounding variables, such as previous experiences with polygynous relationships, familial attitudes toward polygyny, and individual psychological factors such as other personality traits. These unmeasured variables could influence the relationship between attachment styles and emotional responses. Finally, the cross-sectional design of the study limits the ability to draw causal conclusions between attachment styles and emotional reactions. Future longitudinal studies could provide deeper insights into how these relationships may evolve over time and in response to changing circumstances.

By addressing these limitations, future research can build upon this study and further elucidate the intricate relationships between attachment styles, cultural influences, and emotional responses in polygynous marriages.

## 5. Conclusions

To the best of our knowledge, this is the first study to examine the potential individual differences that may influence how young women navigate the stress and resulting negative emotional responses associated with the likelihood of entering a polygynous union. The results of the study may have several important implications for understanding polygamous relationships within Bedouin-Arab culture.

The findings suggest that attachment styles significantly influence how women perceive and react to the threat of polygynous marriages. Women with anxious attachment styles may experience heightened negative emotions such as jealousy and insecurity, while those with avoidant attachment styles may be more tolerant of polygyny. This highlights the need for tailored support and interventions that take individual psychological profiles into account when addressing the challenges faced by women in polygamous settings [2,6,7,9,25]. Additionally, the study underscores the patriarchal nature of Bedouin-Arab society, where polygyny is often examined through the lens of traditional gender roles and social expectations. Understanding the emotional and psychological impacts of polygamous marriages can inform social initiatives aimed at improving the well-being of women in these communities [13]. It emphasizes the importance of addressing the power dynamics and emotional inequalities that can arise in polygynous family structures [6,25]. Furthermore, the emotional distress reported by mothers in polygynous families, as highlighted in previous research, indicates a pressing need for enhanced support systems. This includes mental health resources and community programs that can assist women in navigating the complexities of their family dynamics, coping with feelings of jealousy and rivalry, and fostering resilience [26].

Lastly, the study’s findings may contribute to a broader understanding of how polygamous marriages affect family structures and relationships within Bedouin-Arab communities. By recognizing the psychological impacts of and the strong negative attitudes toward polygyny, stakeholders can better address the social and cultural factors that perpetuate these family dynamics, ultimately leading to more informed policies and practices. Overall, the research highlights the intricate interplay between individual psychological factors and cultural practices, suggesting that a nuanced approach is necessary to support women—particularly anxiously attached women—navigating potential and actual polygamous marriages in Bedouin-Arab society.

## Figures and Tables

**Table 1:** Sociodemographic information.

	Total Sample (*n* = 306)	Low-Threat Condition (*n* = 155)	High-Threat Condition (*n* = 151)
Mean Age	19.68 (2.26)	19.44 (2.12)	19.93 (2.38)
Marital Status			
*Engaged*	7.2%	6.5%	7.9%
*Not Engaged*	92.8%	93.5%	92.1%
Years of Formal Education	12.52 (1.33)	12.37 (1.15)	12.67 (1.48)
Student Status			
*Current Student*	64.1%	64.5%	63.6%
*Not a Student*	35.9%	35.5%	36.4%
Socioeconomic Status			
* Very Good*	16.0%	11.6%	20.5%
* Good*	42.8%	46.5%	39.1%
* Average*	33.0%	34.2%	31.8%
* Bad*	6.5%	7.1%	6.0%
* Very Bad*	1.6%	0.6%	2.6%
Religious Beliefs			
* Religious*	23.5%	25.2%	21.9%
* Traditional*	70.6%	67.7%	73.5%
* Secular*	5.9%	7.1%	4.6%
Family Structure			
* Monogamous*	71.2%	63.9%	78.8%
* Polygynous*	28.8%	36.1%	21.2%

**Table 2:** Intercorrelations and descriptive Statistics.

	1	2	3	4	5
1. Attachment Anxiety	-	0.10	0.54 ***	−0.17 *	0.56 ***
2. Attachment Avoidance	0.13	-	0.09	−0.11	0.24 **
3. Negative Emotional State	0.56 ***	0.32 ***	-	−0.18 *	0.38 ***
4. Polygynous Marriage as a Threat	0.01	−0.15	−0.10	-	0.01
5. Anticipated Negative Emotional Reaction	0.26 **	0.10	0.32 ***	−0.16 *	-
*Mean _Low-Threat Condition_*	3.51	4.04	2.30	6.57	2.09
*Standard Deviation _Low-Threat Condition_*	1.17	0.76	1.06	0.96	1.08
*Skewness _Low-Threat Condition_*	0.15	0.30	0.56	−2.27	1.03
*Kurtosis _Low-Threat Condition_*	−0.16	−0.03	−0.75	4.16	0.33
*Mean _High-Threat Condition_*	3.25	4.04	2.13	6.53	3.09
*Standard Deviation _High-Threat Condition_*	1.14	0.70	1.05	1.09	1.09
*Skewness _High-Threat Condition_*	0.29	0.43	0.76	−2.26	−0.16
*Kurtosis _High-Threat Condition_*	−0.19	0.02	−0.38	3.71	−0.66

The values below the diagonal are taken from participants in the low-threat condition, whereas the values above the diagonal are taken from participants in the high-threat condition. * *p* < 0.05; ** *p* < 0.01; *** *p* < 0.001.

**Table 3:** Comparisons of the low-threat and high-threat conditions.

	Low-Threat Condition (*n* = 155)		High-Threat Condition (*n* = 151)		*t*
	*M*	*SD*	*M*	*SD*	
Attachment Anxiety	3.51	1.17	3.25	1.15	1.92
Attachment Avoidance	4.04	0.76	4.04	0.70	−0.05
Negative Emotional State	2.30	1.06	2.13	1.05	1.42
Polygynous Marriage as a Threat	6.57	0.96	6.53	1.09	0.38
Anticipated Negative Emotional Reaction	2.09	1.08	3.09	1.09	−8.05 ***

*** *p* < 0.001.

**Table 4:** Regression results for perception of polygynous marriage as a threat.

	*B*	*SE*	*t*	*p*	*CI_95%_*
*Step 1*					
Attachment Anxiety	−0.01	0.06	−0.16	0.875	−0.13, 0.11
Attachment Avoidance	−0.15	0.08	−1.84	0.067	−0.31, 0.01
Threat Condition	−0.03	0.06	−0.56	0.578	−0.15, 0.08
Pre-Manipulation Negative Emotional State	−0.11	0.07	−1.65	0.100	−0.24, 0.02
*Step 2*					
Attachment Anxiety × Attachment Avoidance	0.01	0.07	0.17	0.864	−0.13, 0.15
Attachment Anxiety × Threat Condition	−0.09	0.05	−1.75	0.082	−0.19, 0.01
Attachment Avoidance × Threat Condition	0.01	0.08	0.07	0.947	−0.16, 0.17
*Step 3*					
Attachment Anxiety × Attachment Avoidance × Threat Condition	0.06	0.07	0.80	0.423	−0.08, 0.20

Step 1: *R*^2^ = 0.03, *F*(4,301) = 2.46, *p* = 0.046; Step 2: *R*^2^ = 0.04, *F*(7,298) = 1.84, *p* = 0.079; ΔStep 2: Δ*R*^2^ = 0.01, Δ*F*(3,298) = 1.02, *p* = 0.384; Step 3: *R*^2^ = 0.04, *F*(8,297) = 1.69, *p* = 0.100; ΔStep 3: Δ*R*^2^ = 0.00, Δ*F*(1,297) = 0.64, *p* = 0.423.

**Table 5:** Regression results for anticipated negative emotional reaction.

	*B*	*SE*	*t*	*p*	*CI_95%_*
*Step 1*					
Attachment Anxiety	0.29	0.06	5.01	<0.001	0.18, 0.40
Attachment Avoidance	0.14	0.08	1.83	0.069	−0.01, 0.30
Threat Condition	0.55	0.06	9.79	<0.001	0.44, 0.66
Pre-Manipulation Negative Emotional State	0.16	0.07	2.54	0.012	0.04, 0.29
*Step 2*					
Attachment Anxiety × Attachment Avoidance	−0.07	0.07	−1.05	0.294	−0.20, 0.06
Attachment Anxiety × Threat Condition	0.15	0.05	3.05	0.003	0.05, 0.24
Attachment Avoidance × Threat Condition	0.13	0.08	1.62	0.107	−0.03, 0.28
*Step 3*					
Attachment Anxiety × Attachment Avoidance × Threat Condition	−0.08	0.07	−1.16	0.249	−0.21, 0.05

Step 1: *R*^2^ = 0.34, *F*(4,301) = 38.59, *p* < 0.001; Step 2: *R*^2^ = 0.37, *F*(7,298) = 24.78, *p* < 0.001; ΔStep 2: Δ*R*^2^ = 0.03, Δ*F*(3,298) = 4.56, *p* = 0.004; Step 3: *R*^2^ = 0.37, *F*(8,297) = 21.88, *p* < 0.001; ΔStep 3: Δ*R*^2^ = 0.00, Δ*F*(1,297) = 1.34, *p* = 0.249.

## Data Availability

The data presented in this study are available on request from the corresponding authors.
